# “Wire‐Print” as a New Sample Preparation Procedure for Accurate Morphological and Chemical Analysis of Particles and Flakes With Microscopy and Spectroscopy Methods

**DOI:** 10.1002/smtd.70984

**Published:** 2026-08-19

**Authors:** Paul Mrkwitschka, Mario Sahre, Elena Corrao, Francesco Pellegrino, Amaia Zurutuza, Beatriz Alonso, Franziska Emmerling, Vasile‐Dan Hodoroaba

**Affiliations:** ^1^ Division Surface and Thin Film Analysis Bundesanstalt für Materialforschung und ‐prüfung Berlin Germany; ^2^ Department of Chemistry University of Turin Torino Italy; ^3^ Graphenea San Sebastián Spain

**Keywords:** chemical composition, graphene, microscopy, particle size and shape distribution, sample preparation

## Abstract

The newly developed “wire‐print” method is a fast, repeatable and flexible sample preparation procedure aimed at depositing isolated particles or flakes on a substrate for morphological/dimensional analysis, but also for the analysis of the chemical composition. The method was successfully tested on graphene‐related 2D materials (GR2M) and a set of representative reference particles with sizes in nano‐ and micrometer range. Due to their complex morphology and chemistry, the accurate characterization of GR2M is challenging and no standard sample preparation procedure is available. In terms of deposited volume (down to well below 1 nL) and area (down to tens of µm), wire‐print is similar to the piezoelectrical‐driven inkjet printing, but without applying shearing forces to the suspension. It allows for high‐throughput and repeatable deposition of most particulate materials from liquid dispersion as isolated objects onto a substrate. For chemical analysis with Energy‐Dispersive X‐Ray Spectroscopy (EDS) or X‐Ray Photoelectron Spectroscopy (XPS), wire‐printing of µm‐thick and compact spots is also demonstrated. The very short drying times of tiny volumes of suspension imposed by wire‐printing lead to the significant reduction or even disappearance of the typical coffee‐ring effect and thus, to a more homogeneous deposition.

## Graphical abstract

A new method enables reproducible sample preparation via so‐called “wire‐print” deposition. It is characterized by ease of handling and is compatible with most substrates and suspensions. Furthermore, it allows for both the morphological analysis of separated flocs and particles and the chemical examination of compact, thick wire‐print spots.

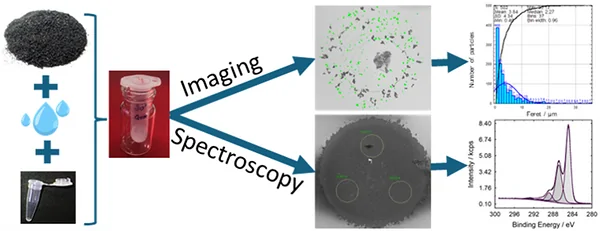

## 1 Introduction

The accurate characterization of commercial graphene‐related 2D materials (GR2M) is challenging [1] due to their complex physico‐chemical properties. Graphene, graphene oxide and GR2M‐containing products have achieved the necessary technical readiness level for commercial use in a variety of application fields [2], since the first sample preparation of single layer graphene was reported [3]. Albeit graphene related products still require further research, the focus has shifted more towards engineering products of sufficient quality and finding new applications [4, 5]. In particle or flake form, GR2M have the tendency to agglomerate, interlock, inter‐twine [6], overlap, crumple [7, 8], fold, etc., when deposited from liquid suspension onto a substrate for subsequent microscopy analysis. To keep the individual flakes as unchanged as possible relative to their original state, significant effort and time must be devoted to optimizing the sample preparation. Depending on each particular material, this could include:

- Ultrasonication (US) of the sample suspension for a given time while introducing a specific energy density into the system.
- Use of suitable dispersing media to stabilize the solids in liquid (organic solvent, water, BSA solution, etc.).
- Tailoring the concentration to enable either the deposition of as many as possible isolated particles/flakes for morphological analysis, or obtaining a compact and thick film for chemical (surface or bulk) analysis.
- Surface pre‐treatment (cleaning, coating, functionalization) of the substrate prior to deposition.

Each of the preparation procedures above should be evaluated ideally by means of quick imaging or/and qualitative chemical analysis. The conventional method to deposit liquid dispersions containing particles or flakes is drop‐casting of a µL‐volume of dispersion onto a suitable substrate (mostly silicon) [9, 10]. The result is a dried sample spot with a lateral size of several mm, typically with an inhomogeneous coverage of the substrate. Generally, it is impractical to drop‐cast samples repeatedly (manually) for purposes of good statistics; also, controlling each intermediate preparation step by imaging is a tedious process. Therefore, several alternative methods such as electrospray ionization deposition [11, 12], dip‐coating [13] or spin coating [14] have been proposed to disperse liquid suspensions (albeit still requiring lengthy pre‐processing steps like US, choice of dispersing agent, etc.) in more or less sophisticated approaches. In the biomedical field, piezoelectric dispensers are used to deposit picolitre (pL) amount of sample on individual windows of a TEM grid [15, 16]. This very precise sample deposition on well‐defined locations on a substrate simplifies considerably the finding of the suitable locations on the deposited sample material for targeted cryogenic TEM imaging. Such optimized sample preparation procedures for the high‐throughput analysis are still lacking for routine SEM analysis of (nano)particulate samples coming from liquid suspension.

In this article we introduce for the first time an own development of a particulate sample deposition method called “wire‐printing” enabling smallest liquid volumes to be deposited on a substrate for subsequent analysis, i. e. droplets smaller than 1 nanoliter (nL), and ending in deposition spots having a diameter down to about 100 µm. The specified dimension is directly determined by the diameter of the wire used as the decisive deposition element for the transfer of the droplet onto a substrate with negligible losses. With this, a large number of mini spots containing samples prepared under various conditions onto a standard substrate of only 1 cm x 1 cm can be easily screened with light microscopy and/or further analyzed in more detail with electron microscopy (SEM or TEM) or other imaging techniques like AFM.

The newly proposed wire‐print deposition procedure is a very promising approach to efficiently achieve optimized deposition qualities (regarding spot homogeneity and substrate coverage degree) as well as the repeatability of results, leading ultimately to better accuracy and reproducibility of the analysis data in general. If for the analysis of size and shape of (nano)particulate matter a wire‐print deposition version is needed to produce basically only single particles deposited homogeneously and at high coverage density on the substrate, for bulk chemical analysis with SEM/EDS it is necessary to apply wire‐print so that a thick (at least a few µm) and compact deposition spot is achieved. Alternatively, a thin (100–200 nm) deposited spot can be also obtained and treated as a thin film for analysis with dedicated X‐microbeam analysis software [17]. For this, dip‐coating or Langmuir‐Blodgett assembly [18, 19] provides reliable ways to homogenously coat films of larger areas on corresponding substrates, with the latter technique resulting in very small thickness.

In this article we demonstrate that wire‐printing has high potential for repeatable deposition of nearly all dispersible particulate samples for both morphological and chemical analysis, while allowing immediate accurate analysis after deposition with less effort in the evaluation/optimization of the sample preparation steps. More specifically, the present study presents wire‐printing as a novel sample preparation method developed and proposed as routine procedure for the accurate physico‐chemical characterization of graphene and graphene oxide flakes dispersed in various media. The analysis methods to be considered are: Light Microscopy (LiMi), Atomic Force Microscopy (AFM), Scanning Electron Microscopy (SEM), Energy‐Dispersive X‐Ray Spectroscopy (EDS), X‐Ray Photoelectron Spectroscopy (XPS), Raman Spectroscopy, Transmission Electron Microscopy (TEM), and so forth. Further, the performance of the new sample preparation approach was tested and validated on ideal monodisperse particulate reference materials.

## 2 Materials

The materials selected for the systematic investigations of the performance of the wire‐print deposition procedure in this article have been focused on GR2M. Further, for the quality control of the newly developed sample preparation approach, several particle reference materials have been selected, prepared according to the same wire‐print deposition procedure, and analyzed.

- First, the 2D materials include graphene flakes dispersed in water with the designation “UT‐G‐01” through “UT‐G‐13” manufactured at the University of Torino with varying synthesis parameters and being referred to in the following as “graphene.”
- Further, commercial graphene oxide flakes dispersed in water and isopropanol (1:1 volume ratio) mixture were studied (Graphenea, Spain).
- As far as the reference particles are concerned, five materials were selected: (i) spherical polymethyl methacrylate (PMMA, 2 µm) (Merck, Germany), (ii) spherical silicon dioxide (250 nm) (Surflay Nanotech, Germany), (iii) spherical silver nanoparticles (6 nm) as a certified reference material (CRM) [20], (iv) colloidal gold nanoparticles (30 nm) as a CRM [21], (v) iron oxide nano‐cubes (8 nm) as a CRM [22].

## 3 Experimental

Sample deposition by wire‐print for the three types of selected materials in liquid suspension was carried out on silicon wafers (with and without a SiO_2_ top layer) for LiMi, SEM, SEM/EDS and XPS analysis as well as on TEM grids (carbon film supported copper grid, standard thickness, grid size 300 mesh) for SEM (working also in the transmission mode) and TEM analysis.

**FIGURE 1:**
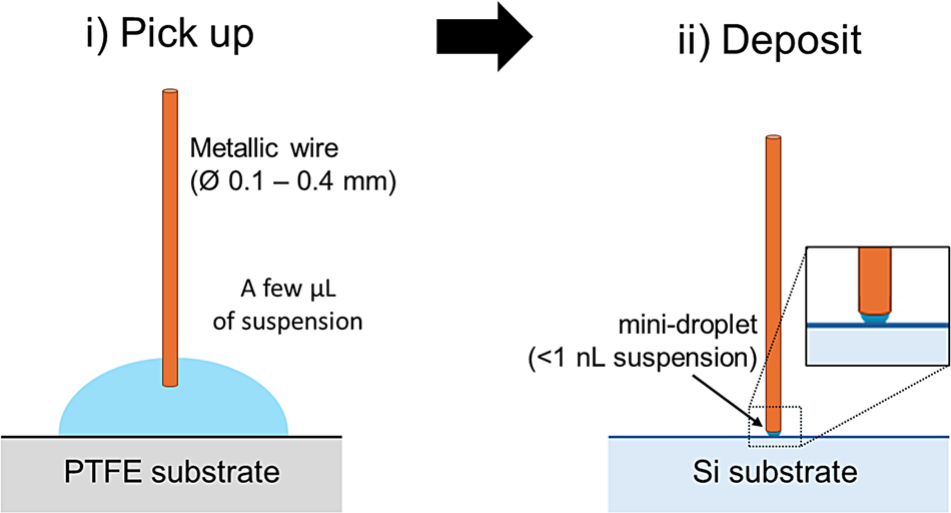
Schematic of the wire‐printing sample preparation procedure.

In the first step, a suspension volume of 0.1 µL to 3 µL, the amount depending on surface tension and volatility of the liquid, is drop‐casted on a 2 mm × 5 mm piece of polytetrafluorethylene (PTFE) foil acting as a reservoir for the following deposition step, see Figure 1. A metallic wire with sufficient stiffness and a diameter ranging from 100 µm (molybdenum) to 500 µm (copper) is dipped with a clean end into the droplet deposited on the PTFE substrate and then retracted. A mini droplet with the volume defined by the wire diameter and its surface energy as well as the surface tension of the liquid remains attached to the wire tip and is “printed” onto a previously cleaned [14] silicon wafer (having a top thin SiO_2_ surface layer), with the wire not touching the substrate, see Figure 1. The wire tip is prepared by grinding to achieve a certain roughness and uniform flatness of its contact surface. We have used a compact manual mechanical XYZ stage (out of a contact resistance meter device) with thread heights of 250 µm for the adjustment in three length coordinates. The visual control by a telescope with 50‐fold magnification enables a positioning precision of less than 20 µm in lateral direction and about 10 µm in height (Z) direction by observing the distance of the wire tip and its mirror‐image in the polished surface of the silicon wafer. After the adjustment of the height, we use a lift‐up‐and‐down mechanism to collect the suspension sample and deposit it in less than 0.5 s. To a certain extent it should be possible to deposit by hand, however, repeatability could suffer substantially and the wires with smaller diameters can deform constantly.

**FIGURE 2:**
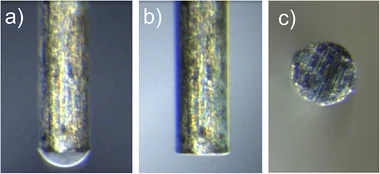
Photographs of the wire (molybdenum, 100 µm diameter) with sample dispersion (a) before the final “print” and (b) dry, after the “print,” and (c) of the tip surface.

To determine the sample amount deposited by wire‐printing, the height, *h*, of the mini‐droplet attached to the wire is measured optically, see Figure 2. With the known wire diameter, *d*, the maximum wire‐printed volume of suspension can be calculated through the equation for a spherical calotte:

(1) $$V=\frac{1}{6}\pi h\cdot \left(3\frac{d^{2}}{4}+h^{2}\right)$$

Thus, for a wire diameter of 100 µm and a height of 30 µm (ultra‐pure water), a deposition volume of 0.13 nL is calculated as a good estimate.

In this article, measurements were performed only by electron microscopy and LiMi with the main purpose of analyzing accurately the particle size and shape distribution by exploiting the wire‐print approach capability to deposit homogeneously isolated particles on a substrate.

A Polyvar‐Met microscope (Reichert‐Jung, Wien) was used to carry out LiMi imaging.

The SEM used to acquire micrographs is of type Supra 40 (Zeiss, Oberkochen, Germany) and is equipped with a high‐resolution Schottky field emitter, a conventional SE (secondary electrons) detector and an SE InLens detector. The measurements were performed at a low beam voltage of 3 and 5 kV in order to keep a superior surface sensitivity for the analysis of the ultra‐thin 2D material flakes.

The captured images were processed using the open access image analysis software package FiJi [23]. Statistical analysis was performed using Origin version 10.0.0.154.

**FIGURE 3:**
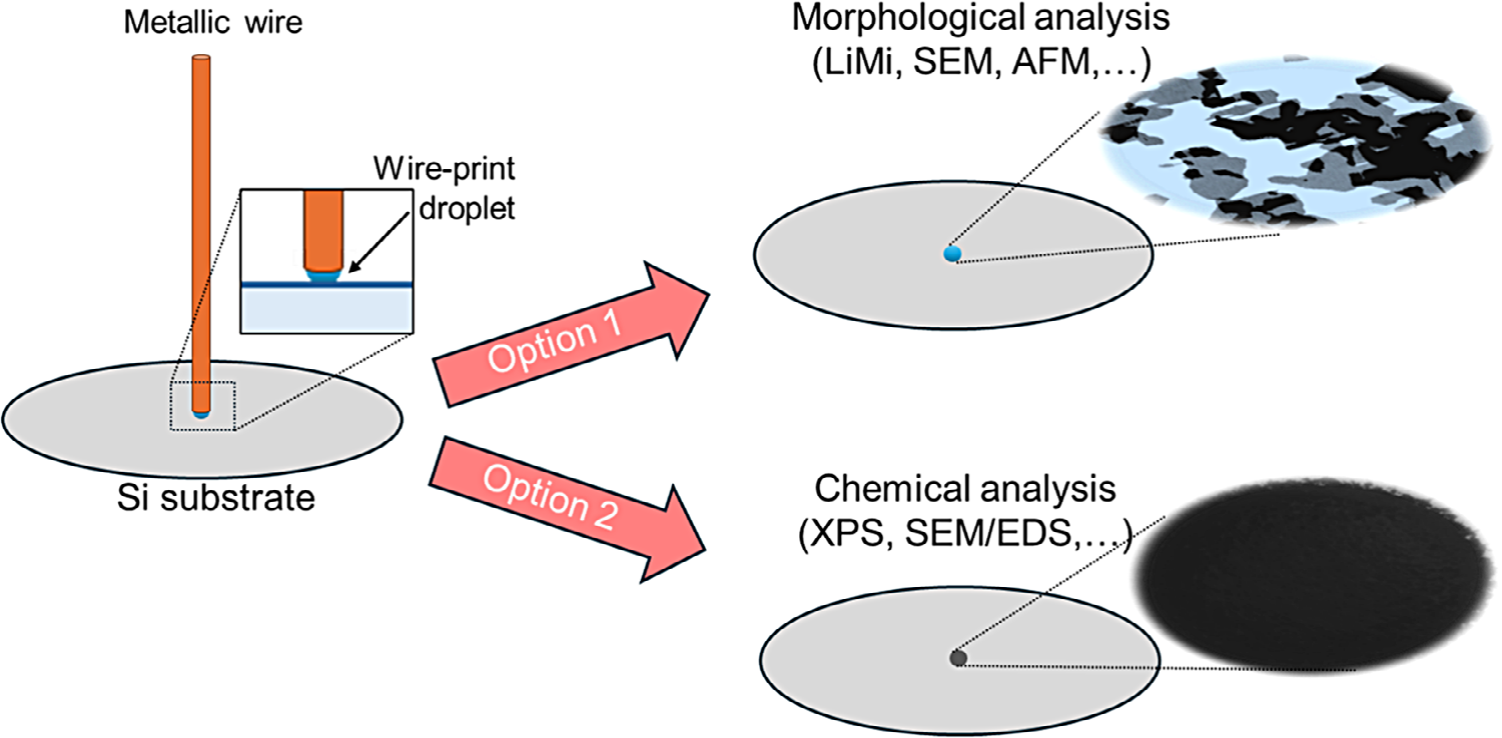
Schematic with the two options of the wire‐print deposition: as low‐concentration particle solution for morphological analysis of individual particles/flakes with microscopy methods (e.g., light microscopy, SEM, AFM) or as thick and compact spot for chemical analysis with spectroscopical methods (SEM/EDS, XPS).

It should be noted that chemical analysis of the same types of particles/flakes in suspension becomes also possible with methods such as SEM/EDS or XPS. However, these methods need a thick and compact deposited spot, which is easily enabled by repeated wire‐print on the same area. The two basic options of the wire‐print deposition are illustrated schematically in Figure 3.

## 4 Results and Discussion

The wire‐print deposition procedure has been applied to graphene and graphene oxide flakes in water‐based suspension, with different deposition parameters on silicon wafer, see Figure 3. Further selected reference particles, i. e. PMMA, Ag, Au, SiO_2_, and Fe_x_O_y_, ranging from µm to nm size, were wire‐printed on the same silicon wafer with the purpose of validating the performance of the new deposition method.

**FIGURE 4:**
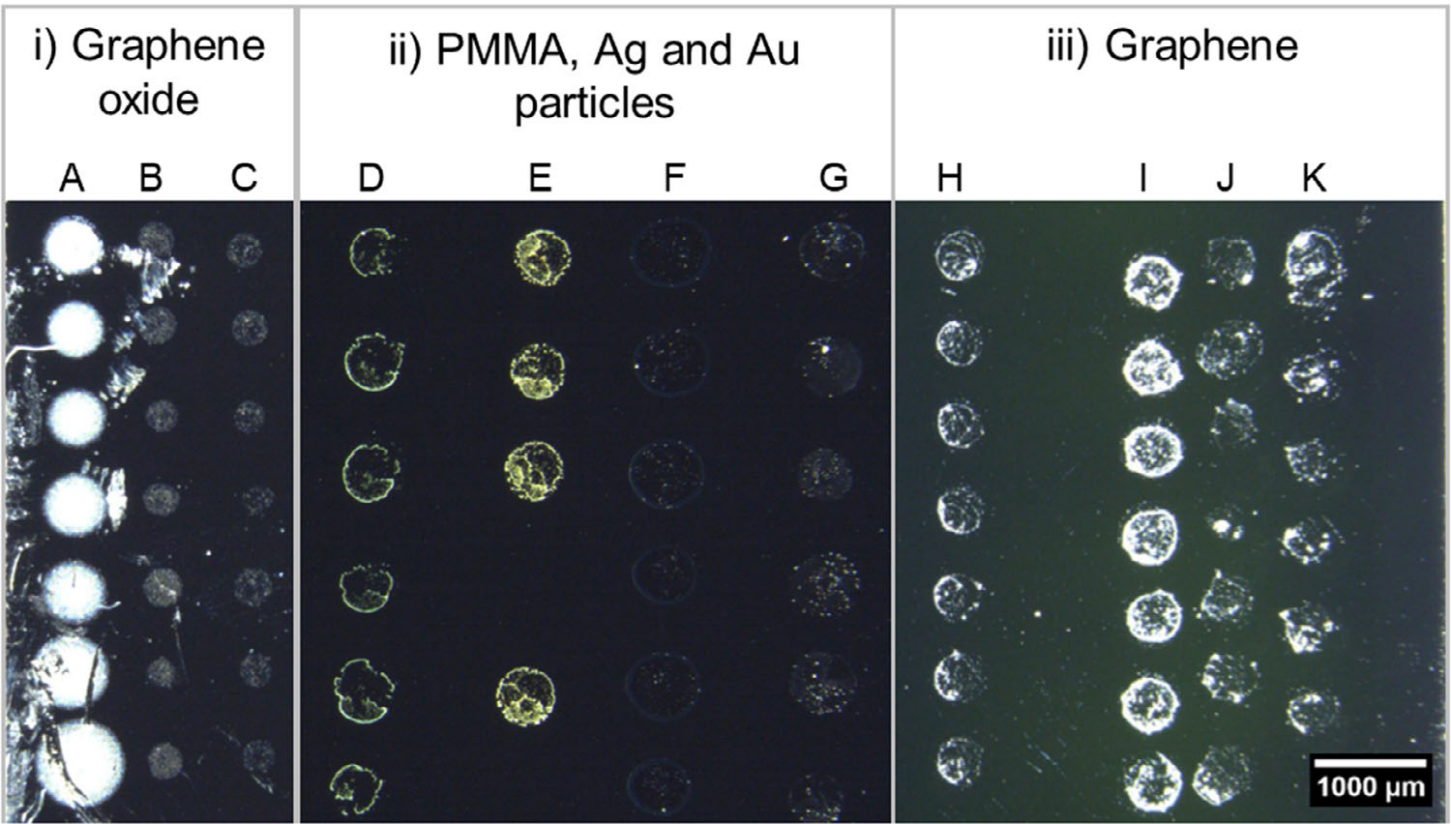
Overview of three different silicon wafer substrates (excerpts) taken with light microscopy, after wire‐printing spots deposited under different conditions, with each column representing one set of same conditions applied repeatedly, for the following materials: (a) commercial one‐layer graphene oxide flakes (columns A to C), (b) reference particles (columns D and E for PMMA micro‐particles, F for Ag nanoparticles, G for Au nanoparticles), and (c) lab‐synthesized graphene (columns H to K). The two missing spots in column E are due to deposition conditions at limit (including operator).

For the graphene and graphene oxide materials, we focus in a first phase on the repeatability of the wire‐print deposition method with regard to the particle size and shape distribution. The deposition repeatability is evaluated, that is, six to eight different spots from each suspension are wire‐printed at different locations on the same substrate (see individual columns in Figure 4) and subsequently analyzed by light microscopy and SEM.

The result of wire‐print preparation of the three selected materials and different deposition conditions is represented as an overview in Figure 4:

- Commercial one‐layer graphene oxide flakes, with column A as the stock solution deposited with 400 µm diameter wire, column B after 1:7 dilution and C after 1:20 dilution of the stock solution from A.
- Nano to micrometer sized particles, with D and E columns representing PMMA micrometer‐particles (1.2 µm nominal diameter in D and 0.5 µm nominal diameter in E), F for Ag nanoparticles, and G for Au nanoparticles.
- Graphene flake suspensions (sample G_01) deposited in the H column with ultrasonic pre‐processing, in I after centrifugal pre‐processing, and in the K and L columns after pre‐processing by shaking.

The best sample preparation approach for sample G_01 was determined and applied to the remaining graphene sample series. As an interesting note, we have observed that, often the first wire‐printed spot differs from the subsequent ones, potentially due to the change from a pristine to the wet surface of the wire. We, therefore, recommend discarding the first spot for quantitative analysis on a case‐by‐case basis.

The graphene oxide material was prepared in two versions for two different purposes: i) deposition of single flakes/particles for the accurate determination of the particle size and shape distribution by SEM, and ii) deposition of thick and compact spots—by variation of the wire diameter and by the dilution of the stock suspension—for the analysis of the chemical composition by EDS and XPS—without (unwanted) substrate contribution.

**FIGURE 5:**
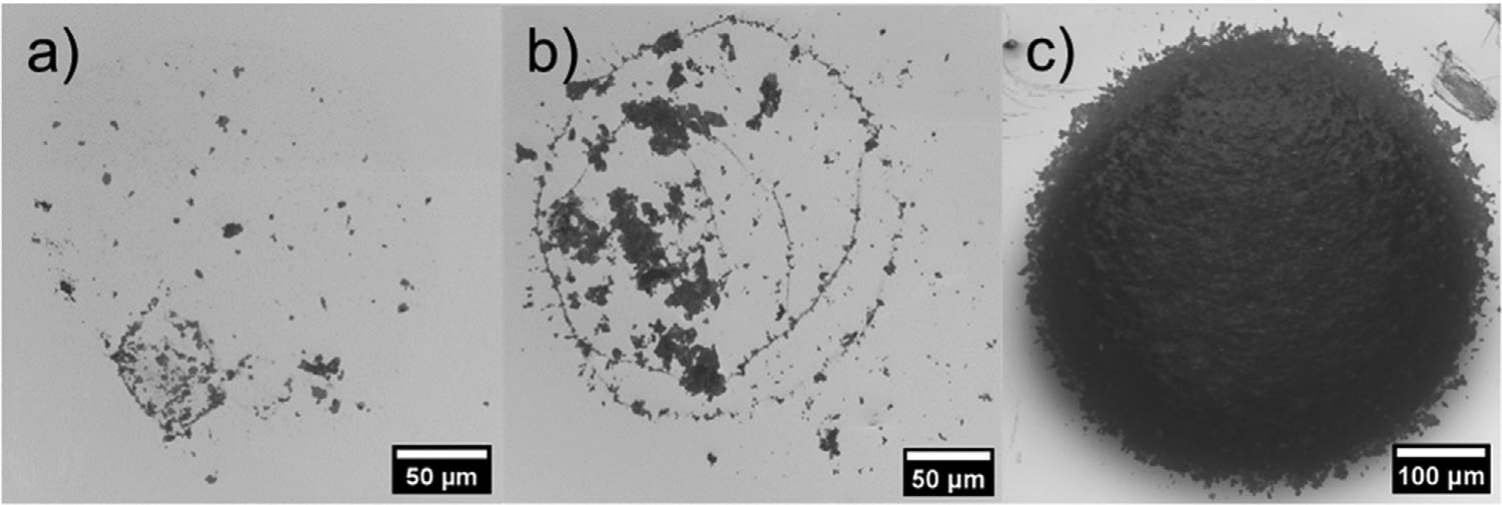
Wire‐print spots of graphene oxide flakes from a water suspension deposited on a silicon substrate: (a) in low concentration of 0.02 g/L for single particle analysis, (b) at intermediate concentration of 0.1 g/L, and (c) as a thick and compact spot using the original stock suspension of 4 g/L for chemical bulk or surface analysis with SEM/EDS and XPS.

Different concentrations of the flakes in water suspension and two different copper wires (diameters of 400 µm and 200 µm) were utilized to tailor accordingly the deposition process, see Figure 5 with representative images. It should be noticed that the formation of intermediate drying artifacts can be observed in Figure 5, which can be explained by the Marangoni effect [24]. Further, the formation of a significant “coffee ring” [25] could be prevented through targeted dilution of the suspension with a suitable solvent and by decreasing the deposited volume.

**FIGURE 6:**
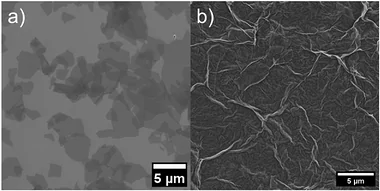
Detailed view of the commercial graphene oxide material prepared by wire‐print from liquid suspension on a silicon substrate as: (a) dispersed individual flakes and (b) a µm‐thick and compact layer.

A detailed representation of the two wire‐print options above is also shown in Figure 6. While Figure 6a shows clearly deposited single and overlapping graphene oxide flakes which are suitable for the determination of the lateral size and shape distribution, Figure 6b visualizes the compactness of the deposited flakes within a compact, thick spot with no substrate being visible.

Note that for the latter deposition option the thickness of the wire‐printed spots is about 0.5 µm, as is the case for the first row “A” shown in Figure 4.

**FIGURE 7:**
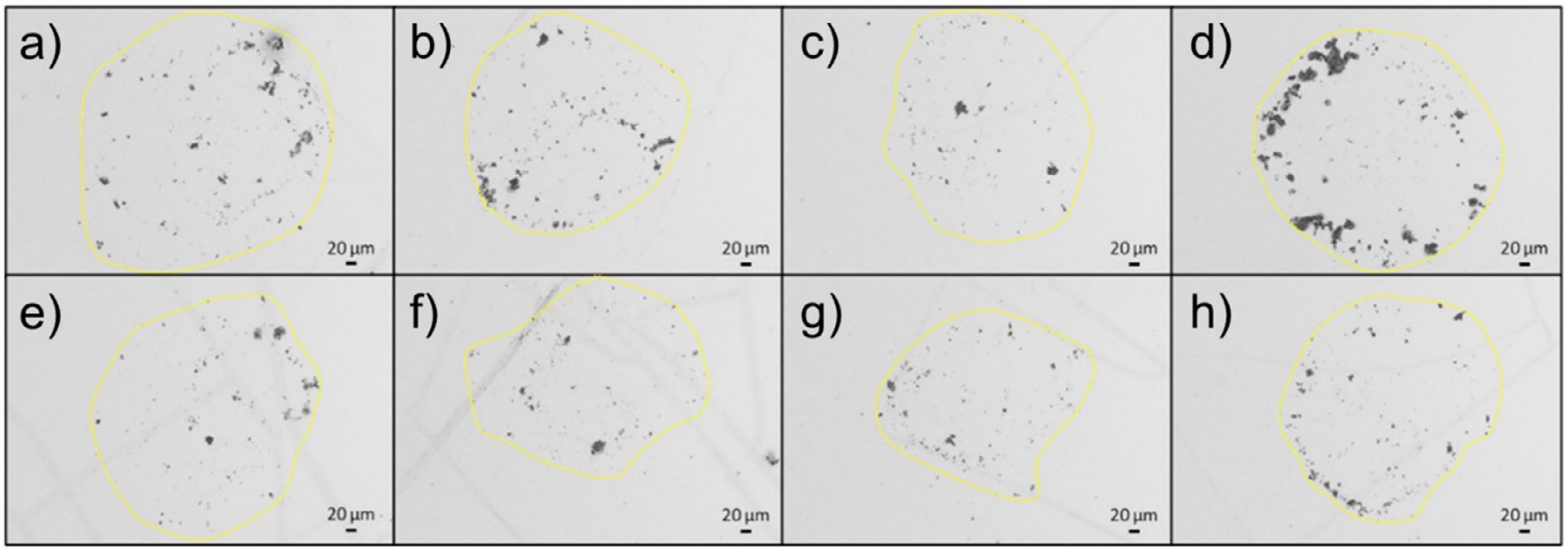
SEM micrographs of wire‐printed graphene flake spots, after repeated wire‐print deposition under the same conditions. For better visibility the spot boundaries have been outlined in yellow.

The wire‐printed spots deposited under the same conditions vary slightly regarding their area and shape. Figure 7 illustrates an example of a series of eight wire‐print depositions of graphene flakes repeated under the same conditions at different locations on the same substrate.

**FIGURE 8:**
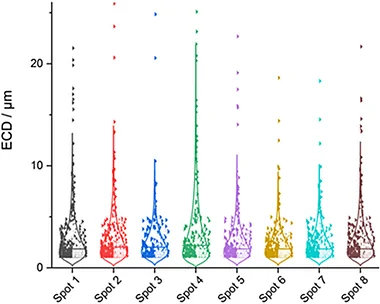
Size distribution of the ECD (Equivalent Circular Diameter) of graphene flakes as calculated from SEM micrographs of eight wire‐printed spots deposited under identical conditions (see Figure 6). The total number of flakes counted within each wire‐printed spot is shown in Table 1.

The lateral size distributions measured on all (!) the graphene flakes deposited within each of the eight wire‐print spots in Figure 7 have been evaluated and the results displayed in Figure 8 and Table 1. All size distributions have maximum sizes ranging up to a few ten of micrometers and, being right‐skewed, were fitted with a lognormal distribution. We chose the equivalent circular diameter (ECD) as a representative size descriptor and the total number of flakes (or particles) deposited within one spot (see Figure 7 and Figure 8, and Table 1). Further morphology descriptors of individual graphene flakes (both constituents and agglomerated/aggregated flakes) for a series of 13 differently synthesized graphene materials are included in the Supporting File, see Figures S1 and S2. The standard deviation values associated with all the morphology descriptors characterizing each wire‐printed spot are also included in Table S1.

**TABLE 1:** Graphene flake size expressed as ECD (Equivalent Circular Diameter) extracted after SEM imaging of the eight wire‐printed graphene flake spots in Figure 7.

Spot number	*N* counted	Mean (µm)	Standard deviation (µm)	Median (µm)
1	333	3.1	3.4	1.8
2	281	3.2	3.7	2.0
3	183	2.7	2.7	2.0
4*	225	4.6*	7.1*	2.2
5	253	2.8	2.8	1.8
6	249	2.5	2.6	1.8
7	271	2.6	2.1	1.8
8	264	2.9	2.8	1.8
Mean	257 (43)	3.1 (0.7)		
RSD	16.9%	22.0%		

*Note*: All flakes deposited within the wire‐printed spots have been counted. With * the size result of the wire‐print spot 4* is marked as deviating from all the other depositions due to some significant agglomeration at the spot boundary (coffee‐ring effect) see Figure 7d. The mean number of flakes counted within all the eight spots is also given together with the mean size accompanied by the standard deviation.

Abbreviation: RSD, relative standard deviation.

To tailor the wire‐print deposition as a thick and compact spot or as individual and well‐dispersed GO flakes within the deposited spot, the concentration of the GO flakes was varied (by dilution of the stock suspension, and with this implicitly the viscosity) and different wire diameters were used, see the deposition result visualized by light microscopy in Figures S3–S7. Also repeated deposition over the same spot can be applied in order to get a thicker deposition, an example is shown in the supporting information Figure S10. For the quantification of the thickness and shape of the wire‐printed spots please refer to the SI, see Figure S8 and Table S2. Further, various fabricated qualities of the morphology of the tip front surface, that is, various roughnesses and tip final shapes, have been tested regarding their effect on the droplet formation, see. Figure S11. Further, particular attention has been paid to the adjustment of the shape of the wire tip by fine processing of its boundary with FIB (Focused Ion Beam), see Figure S12.

Eventually, the thickness of individual flakes occasionally visible as being deposited aside/around the thick wire‐printed spots was determined by AFM, see Figures S9 and S13.

The results in Table 1 demonstrate a good repeatability of the wire‐print deposition with respect to both the lateral flake size distribution and total number of flakes contained in the deposited spot. For the ECD a 22% variance is obtained (as relative standard deviation of the mean value), and for the total number of flakes deposited within each spot a repeatability of 17% (expressed as relative standard deviation) is achieved. One spot only (number 4, see Figure 7d) of the eight is provided with flake agglomeration effects at the spot boundary, that is, the typical coffee‐ring effect. If one excludes this fourth spot of the series of eight, the relative standard deviation decreases from 22% to only 9%.

On a more general note, achieving a high‐quality of the wire‐printed spots is dependent on several factors influencing the deposition from the stock suspension onto the substrate. These include the wire material and its surface tension, the wire diameter or, rather directly related to that, the volume of sample to be deposited, the dispersing media (water, ultra‐pure water, solvent etc.), for example, the correlated vapor pressure, humidity in the laboratory, heating or cooling of the substrate, energy input by centrifugation, ultra‐sonication for deagglomeration purposes, and the applied dilution of the particles or flakes. The observed wire‐printed sample spots exhibit very similar quality with respect to their area and shape.

**FIGURE 9:**
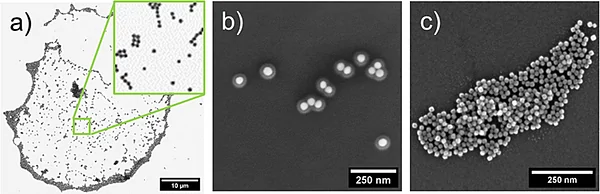
Exemplary wire‐printed sub‐µm and nm particle reference materials: (a) 250 nm SiO_2_ particles on carbon‐coated TEM grid substrate, (b) 30 nm Au nanoparticle certified reference material on silicon wafer, and (c) 8 nm Fe_x_O_y_ nanocubes certified reference material on silicon wafer.

The wire‐print method is applicable not only to GR2M, but also to a variety of nanometer‐ to micrometer‐size particulate materials. So far, we have tested several well‐defined materials such as nano‐Au, nano‐Ag, nano‐iron oxide, sub‐µm SiO_2_ and µm‐size PMMA. Further, the versatility of the wire‐print method makes it possible to deposit particulate materials on most common substrates used in image analysis such as silicon wafers (with and without a top silicon oxide layer) and TEM grids with different coatings, that is, C, SiO_2_, Ni. An overview of a selection of such wire‐printed particle reference materials is shown in Figure 9.

The wire‐printing takes place contactless, with only the liquid droplet touching the substrate surface, so that high repeatability is enabled.

The standard approach when investigating particulate materials in liquid suspension by imaging and spectroscopy techniques is drop‐casting on a substrate with a piston‐operated pipette as an easy‐to‐use, cost‐effective and widespread method. Drop‐casting requires several µL volume of sample which, when applied to a substrate, leads to rather large areas of deposited liquid suspension (several millimeters up to centimeters in size). Wire‐printing offers a cheap and easy‐to‐use alternative, with much smaller volumes of sample dispersion to be applied onto a substrate, that is, nanoliters to picolitres. The wire‐printed sample spots have an area down to 100 µm diameter, that is, orders of magnitude smaller than by drop‐casting. We successfully printed on TEM‐grids as well as on coated and uncoated silicon wafers. Due to the tiny size of the wire‐printed spots, which is defined by the wire diameter, easily in the range of 100 different spots can be wire‐printed on a wafer area of only 1 cm × 1 cm. Thus, a large variability of parameters in sample preparation, that is, different deposition areas, shaking, sonication, sedimentation, and different materials in suspension, can be tested in a row. All the ∼100 wire‐printed spots, including up to 10 repetitions for a set of preparation conditions, can be concentrated on a standard 1 cm^2^ silicon wafer substrate or on a standard TEM grid, which can then be introduced into a SEM analysis chamber only once. One significant benefit of the new wire‐print approach is a huge reduction in the expensive measurement time with advanced instrumentation. Further, due to the high degree of isolated particles deposited within the wire‐printed spots, not only can accurate particle size and shape distributions be measured, but also the total number of deposited particles in the known wire‐printed sample volume is enabled and can be determined, i. e. counted (ideally automated).

As already pointed out above, the other main purpose of wire‐print deposition is the (µm) bulk and surface analysis of the chemical composition by SEM/EDS and XPS, respectively, by means of a µm‐thick and compact spot obtained by repeated wire‐printing on the same area. Representative examples are presented in recent publications of our group, i. e. graphane correlative XPS and EDS analysis in ref. [26] and GO correlative EDS and XPS analysis [26] and [27] are, therefore, not included in this article focused on wire‐print deposition of isolated particles/flakes for the purpose of reliable measurement of the particle/flake size and shape distribution and their number concentration by nano‐imaging methods [28].

Despite these advantages, wire‐printing have some limitations encountered also by other liquid‐based deposition methods and requires a few practical precautions. The quality and homogeneity of the spots are influenced by the solvent volatility and ambient humidity, substrate pre‐treatment and/or surface functionalization, and “cross‐contamination” may occur especially when large particles/agglomerates are deposited in spots being wire‐printed close to each other. Our recommendation is to handle the substrates very carefully, keep the wire tip clean, and use separate wafers for different materials to be wire‐printed.

## 5 Conclusion and Outlook

The new sample preparation approach called “wire‐print” dedicated to the accurate analysis of size and shape as well as chemistry of particulate matter in liquid suspension by microscopy and spectroscopy methods offers clear advantages. Compared to conventional sample deposition methods such as drop‐casting, dip‐coating, spin‐coating, electrospray or piezo‐driven dispensing, wire‐printing combines sub‐nanoliter volumes, negligible shear and a very high density of independent deposition spots on a single substrate in a uniquely simple setup. However, it requires enhanced precision when multiple spots are to be printed with a high density on a reduced area of substrate enabling about 10^2^ distinct spots with different preparation conditions to be deposited on a standard 1‐cm^2^‐substrate, while covering both options of the analytical task to be carried out: isolated flakes for quantitative morphology and compact, micrometer‐thick spots for chemical analysis on the same type of substrate. With the volume of the spots deposited on a substrate by wire‐print being much smaller than those via drop‐cast, that is, in the range of sub‐nL instead of a few µL, the consequence is tiny deposition spots with a diameter defined primarily by diameter of wire used, that is, hundreds of µm. Due to the strongly reduced size of the spots and the resulting much faster drying times, an increased presence of isolated particles or flakes on the substrate can be achieved with superior homogeneity, and, noticeably, without losses. The mostly inherent coffee‐ring effect is either strongly reduced or even not present. Also, the repeatability of the wire‐print deposition is so promising that accurate measurements on particle/flake number concentration by counting them in ideally only one microscopic image become feasible. The successful deposition and analysis of well‐characterized and reference materials combined with reliable metrics for the measurement of size and number concentration of well‐defined flakes, represents a first validation of wire‐printing as a quantitative sample preparation method. These results indicate that wire‐print can reliably deliver both reproducible morphology statistics and controlled sample loadings suitable for spectroscopic techniques. For the purpose of comprehensive method validation, one remaining challenge (subject of a separate study) is the imaging of suitable concentration particle CRMs while simultaneously considering the whole deposited area for the purpose of determining the particle number concentration [29].

Various complexities of flakes and particles have been selected in the present study to test the performance of the newly developed method: commercial graphene oxide flakes, graphene flakes prepared by us and reference particles of different material types, sizes (nano, sub‐µm and µm large) and shapes. To measure the chemical analysis of larger areas and depths of particulate matter from suspension, repeated wire‐print deposition on the same substrate area enables µm‐thick and compact spots, where Energy‐Dispersive X‐Ray Spectroscopy (EDS) with a SEM and/or X‐Ray Photoelectron Spectroscopy (XPS) can be applied, without interfering spectral contributions from the substrate.

The new deposition procedure bears still significant potential for improvement, for example, in the automation of the deposition process. Automated wire‐printing would provide enhanced lateral precision, increased deposition speed and should increase the reproducibility significantly. We are currently exploring the use of an automated stage for high‐throughput and increased quality for the wire‐print sample preparation.

## Conflicts of Interest

The authors declare no conflicts of interest.

## Supporting information




**Supporting File**: smtd70984‐sup‐0001‐SuppMat.docx.

## Data Availability

All data that support the findings of this study are included within the article and supplementary files. Raw data has been uploaded to Zenodo at https://doi.org/10.5281/zenodo.18739384.
