# Supplementary material for: “Wire‐Print” as a New Sample Preparation Procedure for Accurate Morphological and Chemical Analysis of Particles and Flakes With Microscopy and Spectroscopy Methods
Source: Small Methods. 2026 Aug 19;10(18):e70984. doi: 10.1002/smtd.70984 (PMC13599678; doi:10.1002/smtd.70984)
Supplement: Supplementary file 1 [file SMTD-10-e70984-s001.docx]

Electronic Supplementary Material

“Wire-print” as a new sample preparation procedure for accurate morphological and chemical analysis of particles and flakes with microscopy and spectroscopy methods

Paul Mrkwitschka ^a,*^, Mario Sahre ^a^, Elena Corrao ^b^, Francesco Pellegrino ^b^, Amaia Zurutuza ^c^, Beatriz Alonso ^c^, Franziska Emmerling ^a^, Vasile-Dan Hodoroaba ^a,*^

^a^ Bundesanstalt für Materialforschung und -prüfung, Unter den Eichen 87, 12205 Berlin, Germany

^b^ University of Turin, Department of Chemistry, Via Giuria 7, 10125 Torino, Italy

^c^ Graphenea, Paseo Mikeletegi 83, 20009 San Sebastián, Spain

**1. Measurements and descriptors for graphene (UT samples)**....………………………….3

Figure S-1. A) Wire-printed graphene flakes on silicon wafer, B) same as A) with corresponding regions-of-interest (yellow) of the measured particles/flakes …..……………..3

Figure S-2. A) Aspect ratio (AR), B) maximum Feret and C) Solidity of the UT graphene samples extracted from SEM images (e. g. Fig. S-1) after wire-print deposition on silicon wafer………………………………………………………………………...…………………3

Table S-1: Measurands (Mean and Median) extracted from the SEM images for graphene: ECD, maximum Feret, aspect ratio (AR), and solidity, together with respective standard deviations……………………….……………………………………………………………...4

**2. Light Microscopy on GO flakes (Graphenea)**..…………………………………………..5

Figure S-4. Different concentrations of GO flakes deposited with 0.4 mm diameter copper wire………………...…………………………………………………………………………...5

Figure S-5. Different concentrations of GO flakes deposited with 0.4 mm diameter copper wire………………...…………………………………………………………………………...5

Figure S-6. Images of different concentrations GO flakes deposited with 0.25 mm diameter copper wire…………………...………………………………………………………………...6

Figure S-7. GO flakes deposited with copper wires of two diameters: 400 µm (left) and 250 µm (right)…………..…………………………………………………………………………..6

**3. Profilometry on GO flakes (Graphenea)**………………………………………………….6

Figure S-8. Tactile profilometry of selected wire printed spots measured along the marked (orange) line……………………………………………………………………………………7

Table S-2: Mean, standard deviation (SD), maximum thickness and spot diameter of the wire-print GO flake spots measured by tactile profilometry as in Fig. S-8…………………………7

**4. SEM and AFM on GO flakes (Graphenea)**………………………………………………7

Figure S-9. SEM micrograph of a wire-print GO flake spot showing also individual flakes surrounding the spot ...................................................................................................................8

Figure S-10. SEM micrographs of GO flakes: A) deposited in one step, B) after 20 depositions on the same area…………………………………………………………………………...…...8

Figure S-11. Different qualities of the wire tip surface by: i) grinding, ii) fine grinding, iii) polishing, and iv) fine grinding with FIB adjustment of the surface of the wire tip and, finally, of the roundness of the wire tip. Bottom images have captured the corresponding lift-off phase and the first frame after meniscus cutoff……………………………………………………….8

Fig. S-12. SEM micrographs of different quality degrees of the wire tip: i) starting surface, ii) after FIB processing to obtain a round tip surface, iii) after 45 min at 15 nA FIB processing of the entire front area of the tip, iv) after further 45 min at 5 nA FIB processing of the entire front area of the tip, and v) after further 45 min at 15 nA FIB processing of the entire front area of the tip…………………………………………………………………………………...9

Figure S-13. AFM for a GO flake (marked in orange in Fig. S-9) with corresponding height profiles labeled 1 to 3…………………………………………………………………………..9

**1. Measurements and descriptors for graphene (UT samples)**

Fig. S-1 shows the wire-printed graphene flakes on the silicon substrate, well-dispersed, with some agglomerates being present. The marked regions-of-interest (ROIs) show that all particles including agglomerates were measured. The results of 13 different graphene samples are shown in Fig. S-2 and summarized in Table S-1.

**Figure S-1. A) Wire-printed graphene flakes on silicon wafer, B) same as A) with corresponding regions-of-interest (yellow) of the measured particles/flakes.**

**Figure S-2. A) Aspect ratio (AR), B) maximum Feret and C) Solidity of the UT graphene samples extracted from SEM images (e. g. Fig. S-1) after wire-print deposition on silicon wafer.**

The reported measurands ECD, maximum Feret, AR and, additionally, solidity are summarized in Table S-1. The ECD of the 13 GO samples has very similar median values of 0.8 µm with standard deviations ranging from 3.2 µm to 11.6 µm. The maximum Feret has median values between 2.0 µm to 3.9 µm with standard deviations ranging from 5.5 µm to 12.7 µm. All size distributions are right-skewed with maximum values of individual flakes of up to 225 µm. The aspect ratio (AR) corresponding to all materials shows a median value of around 2.0, which indicates a slightly elongated shape. A few flakes for each sample exhibit ratios of up to 8.0 and above. Overall, all samples can be considered similar regarding their AR.

**Table S-1: Measurands (Mean and Median) extracted from the SEM images for graphene: ECD, maximum Feret, aspect ratio (AR), and solidity, together with respective standard deviations**

|  |  | **ECD / µm** | | | **Max Feret / µm** | | | **AR (max 1)** | | | **Solidity (max 1)** | | |
| --- | --- | --- | --- | --- | --- | --- | --- | --- | --- | --- | --- | --- | --- |
|  | *N*  counted | Mean | Median | SD | Mean | Median | SD | Mean | Median | SD | Mean | Median | SD |
| **G_01** | 2067 | 1.9 | 0.8 | 3.2 | 4.8 | 3.0 | 6.2 | 1.9 | 1.9 | 0.6 | 0.82 | 0.8 | 0.14 |
| **G_02** | 513 | 1.7 | 0.8 | 3.7 | 4.4 | 2.7 | 6.3 | 1.95 | 2.0 | 0.6 | 0.81 | 0.8 | 0.15 |
| **G_03** | 895 | 1.9 | 0.8 | 4.6 | 5.5 | 3.0 | 7.8 | 1.9 | 2.0 | 0.6 | 0.80 | 0.8 | 0.15 |
| **G_04** | 594 | 1.9 | 0.8 | 3.6 | 4.4 | 2.7 | 5.5 | 2.0 | 2.0 | 0.8 | 0.81 | 0.8 | 0.15 |
| **G_05** | 678 | 2.2 | 0.8 | 4.2 | 5.3 | 3.3 | 6.4 | 1.9 | 1.9 | 0.6 | 0.79 | 0.8 | 0.15 |
| **G_06** | 500 | 1.9 | 0.8 | 5.0 | 5.1 | 2.8 | 7.4 | 1.9 | 1.8 | 0.6 | 0.80 | 0.8 | 0.14 |
| **G_07** | 360 | 2.0 | 0.8 | 6.7 | 6.7 | 3.1 | 12.7 | 1.9 | 1.7 | 0.6 | 0.80 | 0.8 | 0.15 |
| **G_08** | 1130 | 2.8 | 0.8 | 7.2 | 6.4 | 3.9 | 11.8 | 1.9 | 1.9 | 0.7 | 0.81 | 0.8 | 0.14 |
| **G_09** | 711 | 2.7 | 0.8 | 11.6 | 3.7 | 2.0 | 8.0 | 1.8 | 1.7 | 0.6 | 0.80 | 0.8 | 0.13 |
| **G_10** | 428 | 2.2 | 0.8 | 6.6 | 5.9 | 3.3 | 8.0 | 1.8 | 1.7 | 0.5 | 0.81 | 0.8 | 0.12 |
| **G_11** | 807 | 2.4 | 0.79 | 5.5 | 6.9 | 4.1 | 9.8 | 1.9 | 1.9 | 0.6 | 0.79 | 0.79 | 0.14 |
| **G_12** | 351 | 2.9 | 0.79 | 7.9 | 7.4 | 4.3 | 11.0 | 1.9 | 1.7 | 0.6 | 0.79 | 0.79 | 0.13 |
| **G_13** | 142 | 1.4 | 0.79 | 3.8 | 4.5 | 2.2 | 5.8 | 1.9 | 1.7 | 0.8 | 0.77 | 0.79 | 0.11 |

**2. Light Microscopy on GO flakes (Graphenea)**

Through adjustment of the GO flake concentration and wire diameter it is possible to either deposit individual separated GO flakes or to deposit compact thick GO films.

**Figure S-3. Light microscopy overview image with series of wire-print spots (repeated up to seven times) and the associated sample preparation conditions.**

**0.2 g/l**

**0.5 g/l**

**4 g/l**

**Figure S-4. Different concentrations of GO flakes deposited with 0.4 mm diameter copper wire.**

**0.2 g/l**

**0.5 g/l**

**4 g/l**

**Figure S-5. Different concentrations of GO flakes deposited with 0.4 mm diameter copper wire.**

**0.2 g/l**

**0.5 g/l**

**4 g/l**

**Figure S-6. Images of different concentrations GO flakes deposited with 0.25 mm diameter copper wire.**

**0.4 mm**

**0.25 mm**

**Figure S-7. GO flakes deposited with copper wires of two diameters: 400 µm (left) and 250 µm (right).**

**3. Profilometry on GO flakes (Graphenea)**

All printed spots show the thickest region in the middle of their area, with the thickness decreasing towards the spot boundaries, see Fig. S-8. The average thickness is about 0.4 µm across all measured spots, see Table S-2. The diameter for the shown 7 deposition spots is around 562 µm, with the last two being slightly larger, which leads to a standard deviation of 48 µm (9% relative deviation).

**Figure S-8.** **Tactile profilometry of selected wire-printed GO flake spots measured along the marked (orange) line.**

**Table S-2: Mean, standard deviation (SD), maximum thickness and spot diameter of the wire-print GO flake spots measured by tactile profilometry as in Fig. S-8.**

| **Row 1** | **Mean** | **SD** | **Max.** | **Spot diameter** |
| --- | --- | --- | --- | --- |
|  | **/ µm** | | | |
| P1 | 0.32 | 0.58 | 3.98 | 515 |
| P2 | 0.33 | 0.32 | 1.55 | 507 |
| P3 | 0.29 | 0.30 | 1.41 | 528 |
| P4 | 0.41 | 0.29 | 1.08 | 552 |
| P5 | 0.25 | 0.22 | 1.10 | 570 |
| P6 | 0.50 | 0.32 | 1.28 | 633 |
| P7 | 0.57 | 0.44 | 2.22 | 627 |
| **Mean (all spots)** | **0.4 µm ± 0.1 µm** |  |  | **562 µm ± 48 µm** |

**4. SEM and AFM on GO flakes (Graphenea)**

The deposition of GO flakes by the wire-print method enables AFM analysis. As within the well-dispersed depositions of low-concentrated GO suspensions, monolayers and bilayers were found also on the outskirts of the compact spots, see Fig. S-9 and Fig. S-10.

**Figure S-9. SEM micrograph of a wire-print GO flake spot showing also individual flakes surrounding the spot.**

A

B

**Figure S-10. SEM micrographs of GO flakes: A) deposited in one step, B) after 20 depositions on the same area.**

**Figure S-11. Different qualities of the wire tip surface by: i) grinding, ii) fine grinding, iii) polishing, and iv) fine grinding with FIB adjustment of the surface of the wire tip and, finally, of the roundness of the wire tip. Bottom images have captured the corresponding lift-off phase and the first frame after meniscus cutoff.**

**Fig. S-12. SEM micrographs of different quality degrees of the wire tip: i) starting surface, ii) after FIB processing to obtain a round tip surface, iii) after 45 min at 15 nA FIB processing of the entire front area of the tip, iv) after further 45 min at 5 nA FIB processing of the entire front area of the tip, and v) after further 45 min at 15 nA FIB processing of the entire front area of the tip.**

**Figure S-13. AFM for a GO flake (marked in orange in Fig. S-9) with corresponding height profiles labeled 1 to 3.**
